# Validating an inertial measurement unit for cricket fast bowling: a first step in assessing the feasibility of diagnosing back injury risk in cricket fast bowlers during a tele-sport-and-exercise medicine consultation

**DOI:** 10.7717/peerj.13228

**Published:** 2022-04-07

**Authors:** Keegan Harnett, Brenda Plint, Ka Yan Chan, Benjamin Clark, Kevin Netto, Paul Davey, Sean Müller, Simon Rosalie

**Affiliations:** 1 Curtin School of Allied Health, Curtin University, Perth, Western Australia, Australia; 2 Curtin enAble Institute, Curtin University, Perth, Western Australia, Australia; 3 Curtin School of Nursing, Curtin University, Perth, Western Australia, Australia; 4 School of Science, Psychology and Sport, Federation University, Ballarat, Victoria, Australia

**Keywords:** Wearables, Validation studies, Athletes, Tele-medicine

## Abstract

This study aimed to validate an array-based inertial measurement unit to measure cricket fast bowling kinematics as a first step in assessing feasibility for tele-sport-and-exercise medicine. We concurrently captured shoulder girdle relative to the pelvis, trunk lateral flexion, and knee flexion angles at front foot contact of eight cricket medium-fast bowlers using inertial measurement unit and optical motion capture. We used one sample t-tests and 95% limits of agreement (LOA) to determine the mean difference between the two systems and Smallest Worth-while Change statistic to determine whether any differences were meaningful. A statistically significant (*p* < 0.001) but small mean difference of −4.7° ± 8.6° (95% Confidence Interval (CI) [−3.1° to −6.4°], LOA [−22.2 to 12.7], SWC 3.9°) in shoulder girdle relative to the pelvis angle was found between the systems. There were no statistically significant differences between the two systems in trunk lateral flexion and knee flexion with the mean differences being 0.1° ± 10.8° (95% CI [−1.9° to 2.2°], LOA [−22.5 to 22.7], SWC 1.2°) and 1.6° ± 10.1° (95% CI [−0.2° to 3.3°], LOA [−19.2 to 22.3], SWC 1.9°) respectively. The inertial measurement unit-based system tested allows for accurate measurement of specific cricket fast bowling kinematics and could be used in determining injury risk in the context of tele-sport-and-exercise-medicine.

## Introduction

Cricket fast bowlers have a higher risk of developing low back pain and injury compared to the general athletic population (Stretch, 2003). Factors cited as causative include poor physical preparation and large, acute increases in bowling load (Dennis et al., 2003; Dennis, Finch & Farhart, 2005). Several studies have shown that poor bowling technique is a significant risk factor in the development of back injuries in fast bowlers (Stretch, 2003; Foster et al., 1989; Watkins, 2013). Longitudinal analysis of the incidence of injury in elite cricketers has identified that fast bowlers’ delivery and follow-through (*i.e*., their technique) as “the primary mechanism of injury” (Stretch, 2003). Consequently, it is important to identify fast bowlers whose bowling technique predisposes them to a higher risk of injury.

Fast bowling technique is categorized into four distinct patterns describing the angle between the shoulders and the pelvis relative to the wicket and batsman (Schaefer et al., 2020). The three main fast bowling techniques are: side-on, where the leading hip and shoulder are pointing towards the batsman, the front-on approach, where the hips and shoulder are open and the angle between the wickets and the line joining the shoulders considerably exceeds 180° at rear foot strike (Portus et al., 2004) and a mixed technique, where the lower half of the body is front-on and the upper half side-on to the batsman (Brukner, 2012). The fourth technique known as semi-open, where the upper body position is between front-on and side-on, is sometimes included (Schaefer et al., 2020; Ferdinands et al., 2010). Of these, a mixed bowling technique is the most strongly linked to back injury (Portus et al., 2004) when compared to side-on and front-on bowling techniques (Foster et al., 1989; Portus et al., 2004; Brukner, 2012). This has been attributed to a laterally flexed and hyper-lordotic lumbar spine at front foot contact combined with significantly increased shoulder counter rotation (Foster et al., 1989). Shoulder counter rotation is defined as the amount of axial rotation of the bowling arm shoulder away from the batter between back foot contact and front foot contact during the delivery stride (Ferdinands et al., 2010). Shoulder counter rotation of over 30° is reported to predispose bowlers to back injury (Portus et al., 2004; Ranson et al., 2008). Additionally, bowlers with an extended front knee angle at front foot contact (Crewe et al., 2013) place higher compressive forces through their pars interarticularis (Dennis, Finch & Farhart, 2005). Through cyclical repetitive loading over many overs, a stress response can occur in the vertebra, generally at the pars interarticularis opposite to the bowling arm (Gregory, Batt & Kerslake, 2004; Senington, Lee & Williams, 2018). This repetitive loading may cause a spondylolysis (stress fracture to the pars) or a spondylolisthesis (anterior translation of one vertebra on another) (Gregory, Batt & Kerslake, 2004; Senington, Lee & Williams, 2018). Therefore, its critical to identify bowlers with a mixed bowling action early in their career so that overall exposure to repetitive loading of the pars interarticularis can be reduced.

The traditional method of confirming whether a bowler’s technique includes movement patterns that increase their injury risk, such as a mixed bowling action, is to use optimal motion capture to analyze their kinematics. This is because, like other complex sports actions such as a tennis serve, in the field-setting, a coach is challenged to perceive the kinematics of multiple body segments that are executed at high-speed. It has been reported that even expert coaches with some 15 years of domain experience were not superior to lesser experienced coaches in their accuracy of detecting trunk rotation kinematics in a tennis serve (Giblin et al., 2016). Further, it has been reported that as the degree of trunk rotation increased, accuracy of coach detection of kinematic changes decreased for experienced and less-experienced coaches (Giblin et al., 2016). However, optical motion capture of cricket bowling in a facility that replicates the natural environment and allows bowlers to bowl in a natural manner is typically not feasible in the setting of tele-sport-and-exercise-medicine because it requires either a biomechanics laboratory or for laboratory equipment to be installed within a cricket facility (Worthington, King & Ranson, 2013). Such equipment is expensive and requires extensive training to operate. The laboratory environment itself can hinder bowling technique because it does not accurately replicate a cricket pitch (Dicks, Davids & Button, 2009; Müller, Brenton & Rosalie, 2015). Considerable time is required to both test the athlete and process the resulting data to obtain anatomical angles. Finally, in the post-COVID era it can be extremely difficult for a fast bowler and their coach to access a biomechanics laboratory staffed by specialists with knowledge of cricket bowling required to accurately measure bowling kinematics. Consequently, methods that are as accurate as optical motion capture but more versatile are needed for measuring of bowling kinematics to be feasible in the setting of tele-sport-and-exercise-medicine.

Inertial measurement units (IMUs) promise similar accuracy to laboratory based optical motion capture for the measurement of kinematics, but with much greater usability (Teufl et al., 2019; Beange et al., 2019). These wireless and relatively unobtrusive sensors allow for the analysis of human motion without the need for a laboratory (Camomilla et al., 2018; Ha et al., 2013). IMUs incorporate three types of sensors; an accelerometer, which measures acceleration, a rate gyroscope which measures angular velocity and a magnetometer which measures direction relative to the Earth’s magnetic field. The data from these sensors allows each IMU to measure its kinematics in three dimensions (Camomilla et al., 2018; Ahmad et al., 2013; Elliott, John & Foster, 1989). IMUs digitally filter the data from each sensor to eliminate changes in the variance of the signal (known a drift) (NovAtel, 2014). This helps to eliminate the potential errors and improve the accuracy (Ha et al., 2013; Ahmad et al., 2013; NovAtel, 2014; Roell et al., 2018). Individual IMUs can then be ‘fused’ together to form an array, which provides data in relation to the movements generated between them (Camomilla et al., 2018). This derived data can provide three-dimensional (3D) measurement of multi-body motion such as human movement (Ahmad et al., 2013; Cuesta-Vargas, Galán-Mercant & Williams, 2010). IMUs have been found to accurately measure sports specific movements in ski jumping, netball, rowing, running, tennis, swimming, Australian football and wheelchair rugby (Camomilla et al., 2018). Despite their accuracy and seeming complexity, IMU based motion analysis systems require significantly less training to operate compared to laboratory based optical motion capture. This is because unlike optical motion capture, IMU systems do not require secondary processing of the raw data to generate anatomical angles. Rather, software packaged with commercially available IMU systems generates anatomical angles automatically. These data can be viewed live or downloaded. Therefore, it is feasible for a specialist to remotely analyse bowling technique without physically attending training or competition making IMU systems potentially ideal for tele-sport-and-exercise-medicine.

Here we report the results of a validation of a commercially available IMU system against traditionally accepted methods of assessing bowling technique. The purpose of this research was to evaluate the concurrent validity of a commercially available IMU system against laboratory based optical motion capture for the measurement of cricket fast bowling kinematics. Specifically, the following movements were compared at front foot contact: the amount of axial rotation of the bowling arm shoulder away from the pelvis with respect to the batter (shoulder counter rotation or SCR), front foot knee flexion angle and lateral flexion of the spine away from the bowling arm. We choose to compare these measurements at front foot contact because the combination of significantly increased shoulder counter rotation, lateral flexion of the spine away from the bowling arm and an extended knee at front foot contact have been associated with an increase in the risk of injury to the lumbar spine in fast bowlers (Foster et al., 1989; Ferdinands et al., 2010). We tested the null hypothesis that there would be no difference in these measures between optical motion capture and the commercially available IMU system.

## Materials and Methods

Eight cricket fast bowlers (six right-handed) agreed to participate in this study. Four played at 1st–4th grade district level and four at recreational club level. The mean age of the participants was 26.75 years (SD = 9.16), mean height 181.38 cm (SD = 4.99) and mean mass 81.38 kg (SD = 12.56). Ethical approval from Curtin University’s Human Research Ethics Committee (Approval number HRE2019-0410) was obtained prior to the commencement of testing. Participants were recruited *via* word-of-mouth and through advertisements placed at local cricket clubs. Participants were included in the study if they were over the age of 18, healthy, categorised as ‘fast’ or ‘medium-fast’ bowlers by their respective coaching staff and able to bowl four overs in one continuous session. Exclusion criteria included injury in the past six months that had restricted their ability to bowl during training or matches. We obtained written informed consent from the participants before proceeding with demographic screening using a standardised form.

We used a commercially available, array based IMU system (myoMotion, Noraxon Inc., Scottsdale, AZ, USA). This IMU system uses matchbox sized body-worn sensors to capture 3D kinematics at up to 200 Hz. The configuration can be expanded from two sensors for capturing a single joint of interest to 16 sensors for capturing full-body kinematics. Each sensor is comprised of a triaxial accelerometer with a measurement range of ±16 g, a triaxial gyroscope with a measurement range of ±2,000° s^−1^ and a triaxial magnetometer with a measurement range of ±1.9 Gauss. For this study, five sensors sampling at 200 Hz were used. One was placed at the level of the 7th cervical vertebra (C7) and one at the second sacral vertebra (S2) measured axial rotation of the shoulder girdle relative to the pelvis. The C7 and S2 sensors along with one placed on the humerus of the bowling arm measured lateral flexion of the spine relative to the bowling arm. To measure knee flexion angle one sensor was placed distally on the anterior thigh and one proximally on the anteromedial aspect of the leg. Sensors were located according to the manufacturer specifications for measurement of joint angles between the respective segments. Sensors were attached to the participants using hypoallergenic double-sided medical tape and reinforced with strapping tape. The system was calibrated prior to each data collection using the manufacturer’s process which requires a single click in the software.

An 18-camera maker-based optical motion capture system (Vicon system, Oxford, UK) installed in a motion analysis laboratory was used as the comparator. The optical motion capture system sampled at 250 Hz. Matching the sampling rate of optical motion capture system to that of the IMU system (200 Hz) would adversely affect the accuracy of the optical motion capture system. Instead, optical motion capture data were down sampled to 200 Hz during processing. The system was calibrated prior to testing according to the manufacturer’s specifications. To obtain limb and spinal kinematics, retro-reflective markers were placed at specific landmarks of the participants based on a previously used cricket marker set (Cottam et al., 2018; Cottam et al., 2022). Specifically, single retroreflective markers were placed overlying the suprasternal notch, xiphoid process, the spinous processes of C7 and C10, and bilaterally overlying the anterior superior iliac spine and posterior superior iliac spine. Single retroreflective markers were also placed overlying the anterior and posterior aspects of the glenohumeral joint and the lateral and medial epicondyles of the humerus of the bowling arm. Three marker clusters were placed on the lateral aspect of the bowling arm parallel to the shaft of the humerus and distally 3–4 cm superior to the olecranon as well as overlying the acromion processes bilaterally. Single markers were placed overlying the medial and lateral femoral condyles, the medial malleolus, lateral malleolus, first and fifth metatarsals of the lower limb contralateral to the bowling arm with clusters placed centrally overlying the tibia and posteriorly overlying the calcaneus. An in-ground force plate (AMTI, Watertown, MA, USA) sampling at 2,000 Hz was hardware synchronized to the optical motion capture system to provide precise timing of front foot contact. In addition, both the IMU and optical motion capture systems were hardware synchronized with manufacturer supplied cables to ensure precise time synchronization between the predictor and criterion measures.

To mimic the natural environment and maintain representative task design, we set up the motion analysis laboratory with the key features of a cricket pitch (Müller, Brenton & Rosalie, 2015). We included a strip of artificial turf to replicate a grass surface and reduce the bowlers’ risk of slipping on the force plate. A bowling crease which demarcates the position of the stumps, and a popping crease, which shows where the bowler must place their front foot at ball release, were marked on the artificial turf covering the force plate. A set of stumps were placed to the right of the force plate (for a right-arm bowler). To allow the bowlers to use their normal run-up, the run-through doors were opened. This afforded bowlers approximately 50 m of run-up prior to entering the laboratory. The typical fast bowler’s run-up is between 15–30 m (Elliott, John & Foster, 1989). The bowlers’ task was to bowl at a set of stumps placed 18.9 m from the popping crease at their usual pace and using their usual technique. As the target stumps were outside the laboratory, a standard cricket net was placed across the second run-through door between the force-plate (the bowler’s end) and the target stumps (the batter’s end) to arrest the ball.

Once the participant was prepared for data capture, they were guided through a 15-min warm-up. The warm-up consisted of dynamic stretching and sub-maximal bowling. We then instructed each participant to bowl four six-ball overs at their usual pace. The bowlers were given free choice of line, length, and delivery type. The participants were provided with up to 5-min rest between each over to reduce fatigue.

To allow for a direct comparison between IMU and optical motion capture system, quaternion data was extracted from the IMU system and processed using a custom LabView program. The C7 relative to S2 quaternions were decomposed into ZXY Tait-Bryan angles where X is lateral flexion of the spine, and Y is Axial Rotation (the flexion angle of the spine (Z) was not reported). Likewise, the thigh relative to the shank angle quaternions were decomposed into ZXY Tait-Bryan angles where Z is knee flexion angle. The same decompositions were used to convert marker trajectories from the optical motion capture system to segment angular data using the manufacturer supplied software (Bodybuilder, Vicon system, Oxford, UK) according to previously published methods for assessing kinematics in cricket fast bowling (Cottam et al., 2018). The force plate threshold for initial front foot contact was set at 20N (Cottam et al., 2018). Lastly, a stopwatch was used to measure the time taken to determine the three angles for each method of measurement from initial setup until finalised angle data were available for statistical analysis.

The Bland–Altman method was used to compare the mean difference in axial rotation angle, lateral flexion angle, and knee flexion angle at front foot contact measured by the IMU and optical motion capture systems. In this method the mean difference, which represents the bias between measures, is calculated as the mean of the individual differences (Bland & Altman, 2007). Individual differences are calculated as predicted angle (IMU) minus actual angle (optical motion capture). A one sample t-test with a 95% Confidence Interval (95% CI) was used to test the null hypothesis that the mean difference = 0. Ninety-five percent limits of agreement (95% LOA) were calculated based on the assumption that the true values of axial rotation, lateral flexion, and knee flexion were non-constant between deliveries (Bland & Altman, 2007). In addition, we calculated the smallest worthwhile change as a measure of acceptable variation for field-based measures by multiplying the between-participant standard deviation of the pooled device data by 0.2 (Lacome et al., 2019; Hopkins, 2004; Tran et al., 2010; Wundersitz et al., 2015).

## Results

A total of 192 balls were bowled by the eight bowlers. Occlusion of the optimal markers occurred in 86 balls for axial rotation, 82 balls for lateral flexion and 64 balls for knee flexion. Because this study was examining concurrent validity at a single critical time point, trials were omitted if any marker was occluded at the point of front foot contact. The omission of trials with missing marker data was preferred to performing gap filling by splines in order to maintain data integrity and accuracy by avoiding comparison of inertial motion data to estimated marker positions as opposed to actual marker positions. Hence, 106 balls were anlaysed for agreement of axial rotation, 110 for agreement of lateral flexion and 128 for agreement of knee flexion. G*Power 3.1.9.7 software was used to perform a post-hoc power calculation. This analysis showed that for a sample size of 106 and an alpha level of 0.05 our study had an observed power of 1 to find an effect of size 0.75. This effect size is consistent with previous validations of commercial IMU systems against optical motion capture (Cottam et al., 2022).

**Figure 1:**
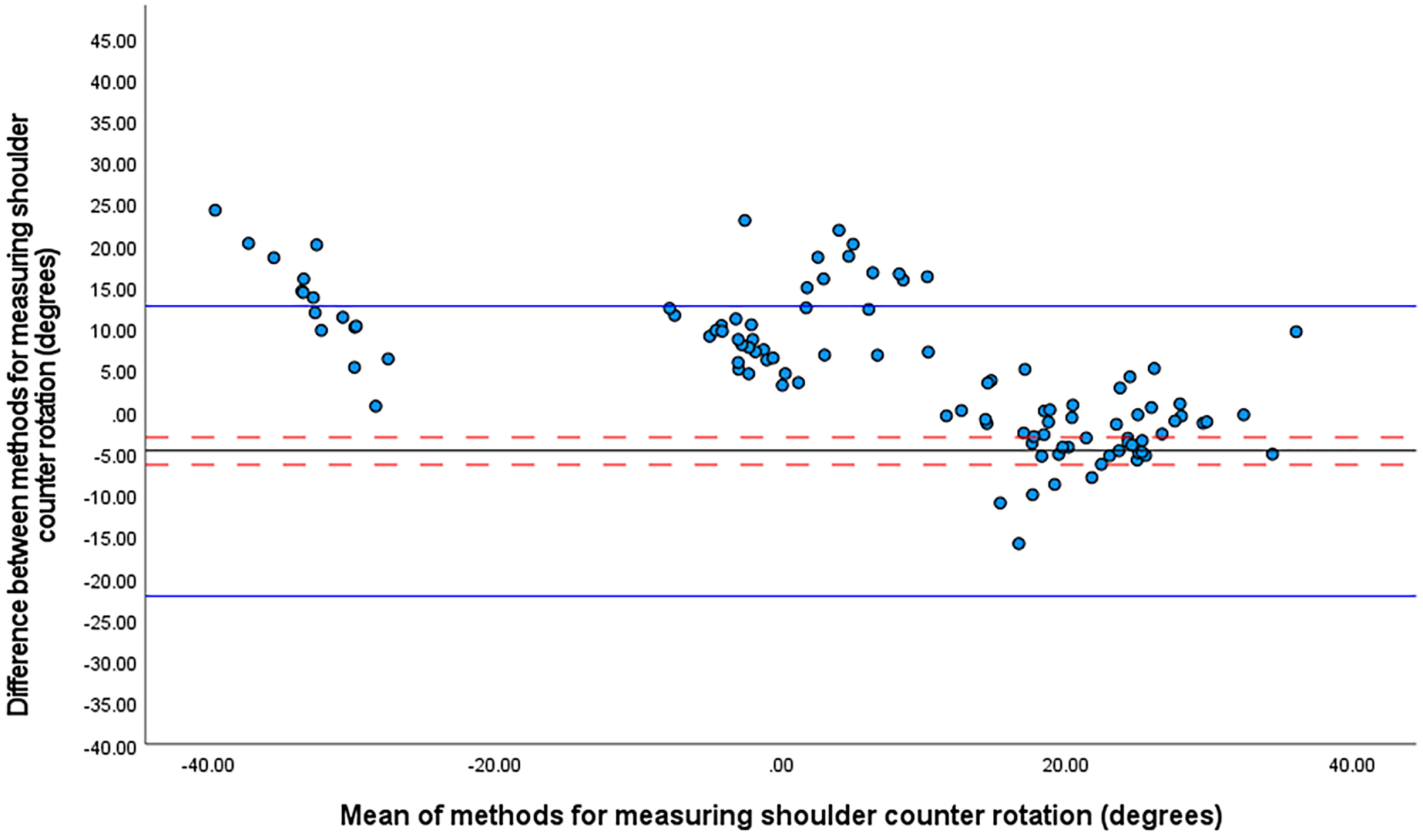
Bland Altman limits of agreement plot for shoulder counter rotation at front foot contact. The mean difference is shown by a solid black line and the upper and lower limits of agreement by solid blue lines. The 95% confidence intervals for each are shown in red dashed lines.

The one sample t-test revealed a significant mean difference between systems in the angle of axial rotation (*p* < 0.001). The axial rotation angle measured by the IMU system was 4.7° ± 8.6° (95% CI [−3.1° to −6.4°], 95% LOA [−22.2° to 12.7°]) less than that measured by optical motion capture (Fig. 1). The smallest worthwhile change was 3.9°.

Figure 1 suggests three distinct patterns of rotation for C7 relative S2 with means centred on 20° (front-on shoulder-girdle and side-on pelvic girdle), 0° (either front-on or side-on) and −30° (side-on shoulder girdle and front-on pelvic girdle).

**Figure 2:**
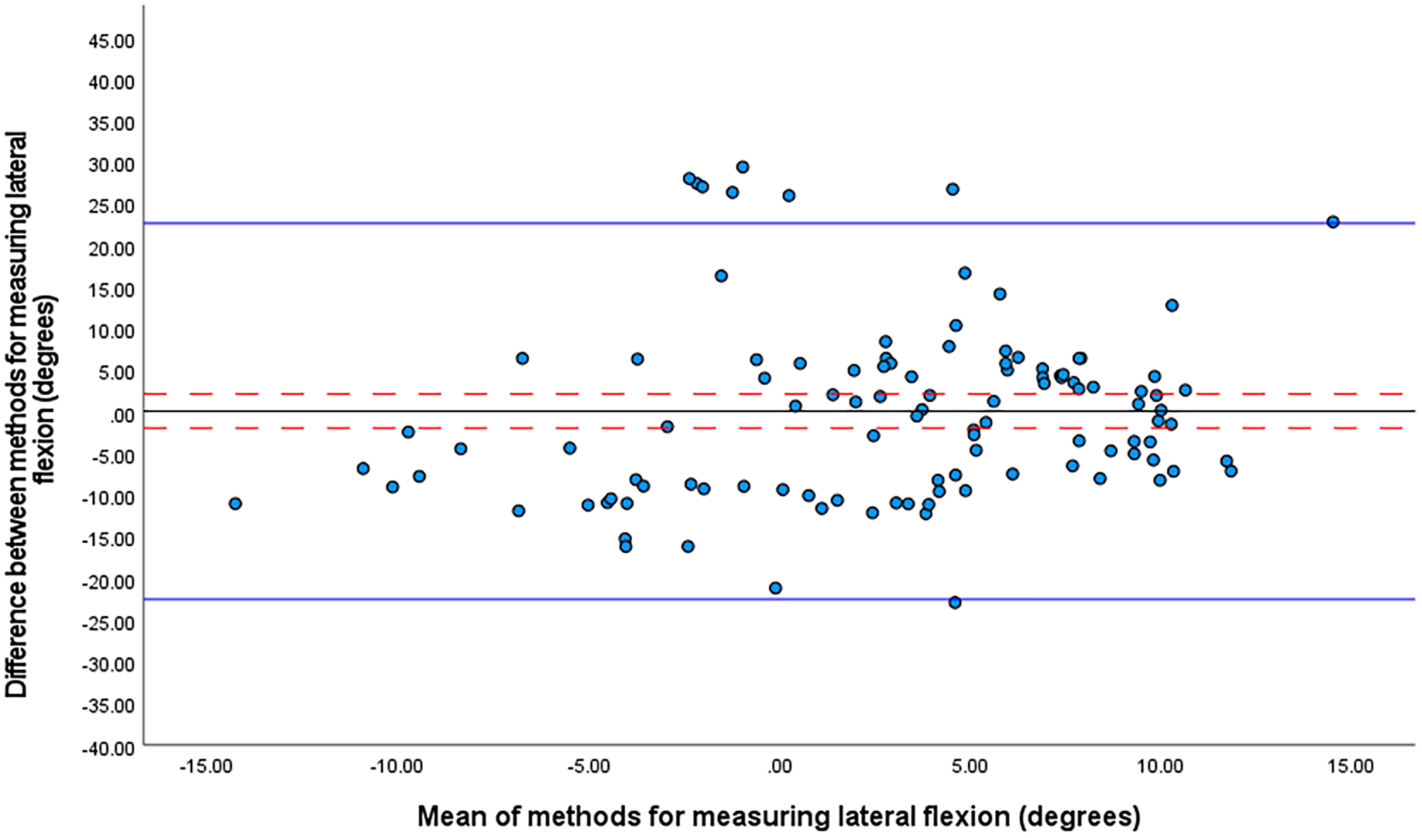
Bland Altman limits of agreement plot for lateral flexion at front foot contact. The mean difference is shown by a solid black line and the upper and lower limits of agreement by solid blue lines. The 95% confidence intervals for each are shown in red dashed lines.

The one sample t-test revealed no significant mean differences between the systems in trunk lateral flexion or knee flexion angles at front foot contact. For trunk lateral flexion, the mean difference between the IMU system and optical motion capture was 0.1° ± 10.8° (95% CI [−1.9 to 2.2°], 95% LOA [−22.5 to 22.7251]) (Fig. 2). The smallest worthwhile change was 1.2°.

**Figure 3:**
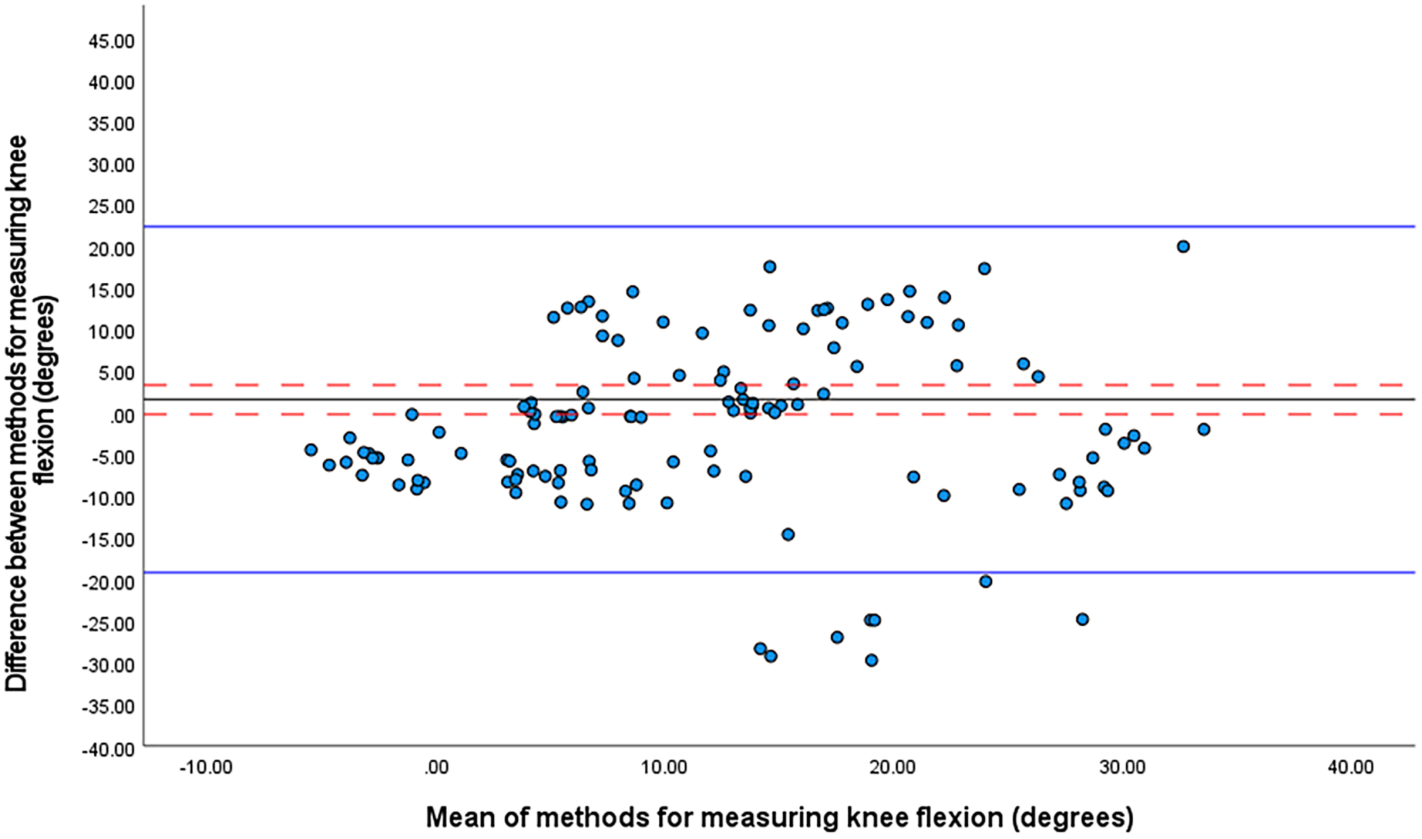
Bland Altman limits of agreement plot for knee flexion at front foot contact. The mean difference is shown by a solid black line and the upper and lower limits of agreement by solid blue lines. The 95% confidence intervals for each are shown in red dashed lines.

The one sample t-test revealed no significant mean differences between the systems in knee flexion angles at front foot contact. For knee flexion, the mean difference was 1.6° ± 10.1° (95% CI [−0.2° to 3.3°], 95% LOA [−19.2 to 22.3]) (Fig. 3). The smallest worthwhile change was 1.9°.

On average, the time taken for initial setup, sensor placement, calibration, and data processing for the IMU system was 30-min. For optical motion capture, an average of 3 h was required for laboratory set up, participant preparation, data processing, data cleaning and extraction. One two-investigator team performed all the data collection. A second two investigator team performed the data processing, cleaning, and extraction. Both teams were equally experienced and trained specifically for this research. It is possible that more experienced investigators could perform these processes more quickly. However, it’s unlikely that even an extensively experienced researcher would be able to collect, process, clean and extract optical motion capture data as quickly as a less experienced researcher would be able to perform an equivalent analysis using a commercial IMU system. This is because the collection processes are simpler for most commercial IMU systems and anatomical angles are generated automatically in real-time.

## Discussion

Our major finding was a small (4.7° ± 8.6°) but significant mean difference for shoulder girdle relative to the pelvis angle between the IMU and optical motion capture systems. Our results are consistent with previous concurrent validity studies comparing IMUs to optically based systems, which have reported a discrepancy of 4° (Godwin, Agnew & Stevenson, 2009; Schall et al., 2016; Poitras et al., 2019). More importantly, although the mean difference was marginally greater than the smallest worthwhile change (3.9°) it was numerically much smaller than the identified threshold for increased injury risk from shoulder counter rotation of 30° (Ranson et al., 2008; Cottam et al., 2018).

Our second finding was that there was no significant mean difference between systems for measurement of trunk lateral flexion at front foot contact. The difference between the IMU and optical motion capture systems was both small (−0.1° ± 10.8°) and less than 10% of the smallest worthwhile change. Similarly small differences for lateral flexion of 1.3°–1.5° (Godwin, Agnew & Stevenson, 2009) and of 0.4°–2.6° (Plamondon et al., 2007) have been reported previously. Obtaining accurate measures of lateral flexion is crucial for the prevention of low back injuries in cricket fast bowling. This is because during front foot contact, lateral flexion is combined with hyper-extension of the lumbar spine and forces of up to 7.3 times body weight can be applied to the facet joints of the lumbar spine (Poitras et al., 2019). These forces have the potential to cause fractures of the pars interarticularis, especially in long durations of bowling (Glazier, 2010).

Our third finding was that there was no significant difference between systems for measurement of when knee flexion angle at front foot contact. The mean difference for knee flexion angle at front foot contact was both small, −1.6° ± 10.1° and less than the smallest worthwhile change (1.9°). This suggests that the IMU systems can accurately measure knee flexion angle for cricket fast bowlers. The mean difference measured in our study was smaller than a previous study which reported a 4° discrepancy between an IMU system and optical motion capture for knee flexion and extension angle during stair ascent (Bergmann, Mayagoitia & Smith, 2009). In cricket fast bowling, having a more extended knee (knee flexion angle < 6.6°) at the front foot contact increases the load on the lumbar spine compared to when the knee is flexed (knee flexion angle > 9.0°) (Crewe et al., 2013). Knee flexion angle plays an important role in attenuating impact force transmitted to the lumbar spine (Elliott, 2000). The small measurement discrepancy between IMU and optically based systems for knee flex-ion angle gives confidence that an IMU system can be used in field-based settings to identify cricket bowlers at higher risk of lumbar spine stress fractures from increased knee extension at front foot contact.

Since concurrent validity of the IMU system has been established, there are several applications of the system such as for bowling technique analysis, as well as skill enhancement in bowlers and coaches. First, the system could be used to screen elite or club bowlers’ techniques in a net practice setting during the pre-season or prior to the start of a tour across geographical locations. This would provide valuable feedback to bowlers and coaches whether for instance shoulder-counter rotation is in a high-risk range. If the action is deemed high-risk, the player and coach could work on a plan to adjust technique in the short-term. This may require consultation with a skill acquisition specialist for guidance in terms of optimal feedback and instruction to re-learn technique (Müller, Fitzgerald & Brenton, 2020) Second, if a bowler’s action is not deemed high-risk of injury, the player and coach could consider the kinematic data for instance to improve bowling speed that might provide a performance advantage. Third, kinematic data from the system of several bowlers’ actions could be used to create point-light displays that presents only the relative motion of white markers on joint centers against a black background (Brenton, Müller & Dempsey, 2019). These point-light displays of various bowlers’ kinematics could be used as visual-perceptual stimuli to train technique identification in coaches from grassroots to professional levels. Point-light displays of a bowler’s action have been used to improve the visual-perceptual skill of highly skilled batters (Brenton, Müller & Dempsey, 2019), so there is potential for the same with coaches. Improved coach detection of bowling actions will provide an initial point of screening with referral for confirmation using the IMU system to guard against injury and/or improve performance.

### Limitations

The major limitation of this study was the use optical motion capture as the reference measure. While optical motion capture is the accepted method and typically considered the “gold standard” for measurement of kinematics in sports biomechanics, it is doubtful whether it is a true criterion measure. Optical motion capture relies on mathematical modelling of markers fixed to skin overlaying anatomical landmarks to obtain joint angles. This introduces two distinct sources of measurement error. One, deformation of the skin and movement of the marker away from the underlying bony landmark, which is considered a critical source of error in optically based methods of estimating joint kinematics (Leardini et al., 2005). Two, the mathematical modelling process (Sezan & Lagendijk, 2012). A recent systematic review has reported that optical motion capture has a discrepancy of 5°–10° when compared to goniometers, inclinometers and radiostereometric measurements (Poitras et al., 2019). However, it is not feasible to use goniometery, inclinometery or radiostereometry in the context of cricket fast bowling. Therefore, the use of optical motion capture as the reference measure in this study is essentially unavoidable.

IMUs suffer from similar issues with measurement error due to skin and superficial tissue movement to optical motion capture. However, unlike optical motion capture, joint angles are determined directly based on the movement of one unit relative to another rather than modelled on movement of subcutaneous landmarks. Nonetheless, accurate placement of the sensors is necessary to avoid misalignment between the coordinate systems of the joint and the sensor (Seel, Raisch & Schauer, 2014). This could potentially limit the use of IMU systems by coaches and athletes who may have limited knowledge of surface anatomy. An effective calibration procedure is necessary to reduce the issues with misalignment of sensor and joint coordinate systems (Kianifar et al., 2019). Several IMU systems have been able to achieve this so that placement site does not adversely affect measurement accuracy. However, not all IMU systems have calibration procedures that are sufficiently robust to overcome issues with misalignment (Kianifar et al., 2019; Horsley et al., 2021) and some manufacturers suggest that calibration is unnecessary (de Fontenay et al., 2020). In addition, some systems may be more sensitive to locating sensors in positions other than the manufacturer’s recommended sites (Brice, Hurley & Phillips, 2018). Moreover, calibration procedures for some systems are not “inbuilt” as part of the manufacturer’s software instead requiring additional equipment and technical knowledge (Kianifar et al., 2019). Therefore, potential users such as coaches or practitioners should be advised to carefully select an IMU system that has an inbuilt calibration procedure that is sufficiently robust to avoid systematic measurement errors from misalignment between coordinate frames and variation in sensor placement.

## Conclusions

IMU systems are a more feasible solution compared to optical motion capture for measuring the kinematics of fast bowlers in the setting of tele-sport-and-exercise-medicine. IMU systems can provide near real-time feedback from measurements taken during a practice session. In our study, results from the IMU system were available in 30 min compared to 3 h for optical motion capture. Therefore, with an IMU system it is feasible for a bowler’s injury risk to be compared between their baseline technique and a modified technique within a single 60-min practice session. It is impossible to perform a similar assessment using laboratory based optical motion capture. In addition, unlike the optical motion capture system, IMU systems with robust calibration procedures do not require in-depth knowledge of surface anatomy to attach the sensors or technical knowledge to calibrate the system. This makes remote diagnosis based on live or down-loaded data possible, which is critical when specialist diagnosticians and/or laboratory services are not locally available to players and coaches. Therefore, kinematic analysis of fast bowling technique in a tele-sport-and-exercise medicine consultation is feasible with IMU based motion analysis but not optical motion capture.

## Supplemental Information

10.7717/peerj.13228/supp-1 Supplemental Information 1 Extracted IMU and optical motion capture mechanics synchronised *via* the force plate.

## Additional Information and Declarations

### Data Availability

The following information was supplied regarding data availability: The raw data is available in the Supplemental File.

## References

[ref-1] Ahmad N, Ghazilla RAR, Khairi NM, Kasi V (2013). Reviews on various inertial measurement unit (IMU) sensor applications. International Journal of Signal Processing Systems.

[ref-2] Beange KH, Chan AD, Beaudette SM, Graham RB (2019). Concurrent validity of a wearable IMU for objective assessments of functional movement quality and control of the lumbar spine. Journal of Biomechanics.

[ref-3] Bergmann JH, Mayagoitia RE, Smith IC (2009). A portable system for collecting anatomical joint angles during stair ascent: a comparison with an optical tracking device. Dynamic Medicine.

[ref-4] Bland JM, Altman DG (2007). Agreement between methods of measurement with multiple observations per individual. Journal of Biopharmaceutical Statistics.

[ref-5] Brenton J, Müller S, Dempsey A (2019). Visual-perceptual training with acquisition of the observed motor pattern contributes to greater improvement of visual anticipation. Journal of Experimental Psychology: Applied.

[ref-6] Brice SM, Hurley M, Phillips EJ (2018). Use of inertial measurement units for measuring torso and pelvis orientation, and shoulder-pelvis separation angle in the discus throw. International Journal of Sports Science & Coaching.

[ref-7] Brukner P (2012). Brukner & Khan’s clinical sports medicine.

[ref-8] Camomilla V, Bergamini E, Fantozzi S, Vannozzi G (2018). Trends supporting the in-field use of wearable inertial sensors for sport performance evaluation: a systematic review. Sensors.

[ref-9] Cottam DS, Campbell AC, Davey MPC, Kent P, Elliott BC, Alderson JA (2022). Measurement of uni-planar and sport specific trunk motion using magneto-inertial measurement units: the concurrent validity of Noraxon and Xsens systems relative to a retro-reflective system. Gait & Posture.

[ref-10] Cottam D, Steven K, Amity C, Paul D, Peter K, Jay-Shian T, Bruce E, Jacqueline A (2018). Can inertial measurement units be used to validly measure pelvis and thorax motion during cricket bowling?. ISBS Proceedings Archive.

[ref-11] Crewe H, Campbell A, Elliott B, Alderson J (2013). Lumbo-pelvic loading during fast bowling in adolescent cricketers: the influence of bowling speed and technique. Journal of Sports Sciences.

[ref-12] Cuesta-Vargas AI, Galán-Mercant A, Williams JM (2010). The use of inertial sensors system for human motion analysis. Physical Therapy.

[ref-13] de Fontenay BP, Roy JS, Dubois B, Bouyer L, Esculier JF (2020). Validating commercial wearable sensors for running gait parameters estimation. IEEE Sensors Journal.

[ref-14] Dennis R, Farhart R, Goumas C, Orchard J (2003). Bowling workload and the risk of injury in elite cricket fast bowlers. Journal of Science and Medicine in Sport.

[ref-15] Dennis R, Finch CF, Farhart P (2005). Is bowling workload a risk factor for injury to Australian junior cricket fast bowlers?. British Journal of Sports Medicine.

[ref-16] Dicks M, Davids K, Button C (2009). Representative task design for the study of perception and action in sport. International Journal of Sport Psychology.

[ref-17] Elliott BC (2000). Back injuries and the fast bowler in cricket. Journal of Sports Sciences.

[ref-18] Elliott BC, John D, Foster DH, Elliott B, Foster D, Blanksby B (1989). Factors which may predispose a bowler to injury. Send the Stumps Flying: The Science of Fast Bowling.

[ref-19] Ferdinands R, Kersting UG, Marshall RN, Stuelcken M (2010). Distribution of modern cricket bowling actions in New Zealand. European Journal of Sport Science.

[ref-20] Foster D, John D, Elliott B, Ackland T, Fitch K (1989). Back injuries to fast bowlers in cricket: a prospective study. British Journal of Sports Medicine.

[ref-21] Giblin G, Farrow D, Reid M, Ball K, Abernethy B (2016). Does perceptual or motor experience influence the perception of global and joint-specific kinematic changes in complex movement patterns?. Attention, Perception, & Psychophysics.

[ref-22] Glazier PS (2010). Is the ‘crunch factor’an important consideration in the aetiology of lumbar spine pathology in cricket fast bowlers?. Journal of Sports Medicine.

[ref-23] Godwin A, Agnew M, Stevenson J (2009). Accuracy of inertial motion sensors in static, quasistatic, and complex dy-namic motion. Journal of Biomechanical Engineering.

[ref-24] Gregory P, Batt M, Kerslake R (2004). Comparing spondylolysis in cricketers and soccer players. British Journal of Sports Medicine.

[ref-25] Ha T-H, Saber-Sheikh K, Moore AP, Jones MP (2013). Measurement of lumbar spine range of movement and coupled motion using inertial sensors-a protocol validity study. Manual Therapy.

[ref-26] Hopkins WG (2004). How to interpret changes in an athletic performance test. Sportscience.

[ref-27] Horsley BJ, Tofari PJ, Halson SL, Kemp JG, Dickson J, Maniar N, Cormack SJ (2021). Impact of inertial measurement unit placement on the validity and reliability of stride variables during running: a systematic review and meta-analysis. Sports Medicine.

[ref-28] Kianifar R, Joukov V, Lee A, Raina S, Kulić D (2019). Inertial measurement unit-based pose estimation: analyzing and reducing sensitivity to sensor placement and body measures. Journal of Rehabilitation and Assistive Technologies Engineering.

[ref-29] Lacome M, Peeters A, Mathieu B, Bruno M, Christopher C, Piscione J (2019). Can we use GPS for assessing sprinting performance in rugby sevens? A concurrent validity and between-device reliability study. Biology of Sport.

[ref-30] Leardini A, Chiari L, Croce UD, Cappozzo A (2005). Human movement analysis using stereophotogrammetry: Part 3. Soft tissue artifact assessment and compensation. Gait & Posture.

[ref-31] Müller S, Brenton J, Rosalie SM (2015). Methodological considerations for investigating expert interceptive skill in in situ settings. Sport, Exercise, and Performance Psychology.

[ref-32] Müller S, Fitzgerald C, Brenton J (2020). Considerations for application of skill acquisition in sport: an example from tennis. Journal of Expertise.

[ref-33] NovAtel (2014). IMU errors and their effects. https://www.novatel.com/assets/Documents/Bulletins/APN064.pdf.

[ref-34] Plamondon A, Delisle A, Larue C, Brouillette D, McFadden D, Desjardins P, Larivière C (2007). Evaluation of a hybrid system for three-dimensional measurement of trunk posture in motion. Applied Ergonomics.

[ref-35] Poitras I, Dupuis F, Bielmann M, Campeau-Lecours A, Mercier C, Bouyer L, Roy J-S (2019). Validity and reliability of wearable sensors for joint angle estimation: a systematic review. Sensors.

[ref-36] Portus MR, Mason BR, Elliott BC, Pfitzner MC, Done RP (2004). Cricket: technique factors related to ball release speed and trunk injuries in high performance cricket fast bowlers. Sports Biomechanics.

[ref-37] Ranson CA, Burnett AF, King M, Patel N, O’Sullivan PB (2008). The relationship between bowling action classification and three-dimensional lower trunk motion in fast bowlers in cricket. Journal of Sports Sciences.

[ref-38] Roell M, Roecker K, Gehring D, Mahler H, Gollhofer A (2018). Player monitoring in indoor team sports: concurrent validity of inertial measurement units to quantify average and peak acceleration values. Frontiers in Physiology.

[ref-39] Schaefer A, Ferdinands RED, O’Dwyer N, Edwards S (2020). A biomechanical comparison of conventional classifications of bowling action-types in junior fast bowlers. Journal of Sports Sciences.

[ref-40] Schall MC, Fethke NB, Chen H, Oyama S, Douphrate DI (2016). Accuracy and repeatability of an inertial measurement unit system for field-based occupational studies. Ergonomics.

[ref-41] Seel T, Raisch J, Schauer T (2014). IMU-based joint angle measurement for gait analysis. Sensors.

[ref-42] Senington B, Lee RY, Williams JM (2018). Ground reaction force, spinal kinematics and their relationship to lower back pain and injury in cricket fast bowling: a review. Journal of Back and Musculoskeletal Rehabilitation.

[ref-43] Sezan MI, Lagendijk RL (2012). Motion analysis and image sequence processing.

[ref-44] Stretch R (2003). Cricket injuries: a longitudinal study of the nature of injuries to South African cricketers. British Journal of Sports Medicine.

[ref-45] Teufl W, Miezal M, Taetz B, Fröhlich M, Bleser G (2019). Validity of inertial sensor based 3D joint kinematics of static and dynamic sport and physiotherapy specific movements. PLOS ONE.

[ref-46] Tran J, Netto K, Aisbett B, Gastin P (2010). Validation of accelerometer data for measuring impacts during jumping and landing tasks.

[ref-47] Watkins L (2013). Movement variability and its relationship to injury mechanisms within the fast bowling action in cricket. https://repository.cardiffmet.ac.uk/handle/10369/4898.

[ref-48] Worthington PJ, King MA, Ranson CA (2013). Relationships between fast bowling technique and ball release speed in cricket. Journal of Applied Biomechanics.

[ref-49] Wundersitz DWT, Gastin PB, Richter C, Robertson SJ, Netto KJ (2015). Validity of a trunk-mounted accelerometer to assess peak accelerations during walking, jogging and running. European Journal of Sport Science.

